# Hinokitiol Induces DNA Damage and Autophagy followed by Cell Cycle Arrest and Senescence in Gefitinib-Resistant Lung Adenocarcinoma Cells

**DOI:** 10.1371/journal.pone.0104203

**Published:** 2014-08-08

**Authors:** Lan-Hui Li, Ping Wu, Jen-Yi Lee, Pei-Rong Li, Wan-Yu Hsieh, Chao-Chi Ho, Chen-Lung Ho, Wan-Jiun Chen, Chien-Chun Wang, Muh-Yong Yen, Shun-Min Yang, Huei-Wen Chen

**Affiliations:** 1 Graduate Institute of Toxicology, College of Medicine, National Taiwan University, Taipei, Taiwan; 2 Department of Laboratory, Kunming Branch, Taipei City Hospital, Taipei, Taiwan; 3 Department of Internal Medicine, National Taiwan University Hospital and National Taiwan University Medical College, Taipei, Taiwan; 4 Division of Wood Cellulose, Taiwan Forestry Research Institute, Taipei, Taiwan; 5 Graduate Institute of Oncology, College of Medicine, National Taiwan University, Taipei, Taiwan; 6 Division of Infectious Diseases, Kunming Branch, Taipei City Hospital, Taipei, Taiwan; 7 Department of Medicine, National Yang-Ming University, Taipei, Taiwan; 8 Department of Pathology, Tri-Service General Hospital, National Defense Medical Center, Taipei, Taiwan; Columbia University Medical Center, United States of America

## Abstract

Despite good initial responses, drug resistance and disease recurrence remain major issues for lung adenocarcinoma patients with epidermal growth factor receptor (EGFR) mutations taking EGFR-tyrosine kinase inhibitors (TKI). To discover new strategies to overcome this issue, we investigated 40 essential oils from plants indigenous to Taiwan as alternative treatments for a wide range of illnesses. Here, we found that hinokitiol, a natural monoterpenoid from the heartwood of *Calocedrus formosana*, exhibited potent anticancer effects. In this study, we demonstrated that hinokitiol inhibited the proliferation and colony formation ability of lung adenocarcinoma cells as well as the EGFR-TKI-resistant lines PC9-IR and H1975. Transcriptomic analysis and pathway prediction algorithms indicated that the main implicated pathways included DNA damage, autophagy, and cell cycle. Further investigations confirmed that in lung cancer cells, hinokitiol inhibited cell proliferation by inducing the p53-independent DNA damage response, autophagy (not apoptosis), S-phase cell cycle arrest, and senescence. Furthermore, hinokitiol inhibited the growth of xenograft tumors in association with DNA damage and autophagy but exhibited fewer effects on lung stromal fibroblasts. In summary, we demonstrated novel mechanisms by which hinokitiol, an essential oil extract, acted as a promising anticancer agent to overcome EGFR-TKI resistance in lung cancer cells via inducing DNA damage, autophagy, cell cycle arrest, and senescence in vitro and in vivo.

## Introduction

Lung cancer, especially lung adenocarcinoma [1], is the leading cause of cancer mortality worldwide, with a five-year overall survival rate of only 15% [2], [3]. Clinical and epidemiologic evidence has shown that more than 50% of lung adenocarcinoma cases in Asia express the epidermal growth factor receptor (EGFR) mutation. Accordingly, EGFR-tyrosine kinase inhibitors (TKIs) have been developed and have been shown to improve survival over standard treatments [4], [5]. Despite good initial responses to EGFR-TKIs, most lung adenocarcinoma patients with EGFR mutations who are taking EGFR-TKIs develop resistance within 9 months. Resistance may be due to either intrinsic or acquired tumor cell resistance to both conventional and targeted cancer therapies and remains one of the largest clinical obstacles [6]. Therefore, there is an urgent need to identify new strategies for the treatment of lung cancer patients with drug resistance.

Herbal compounds have been suggested as an important and classical source for developing strategies to treat cancers. In folk medicine, essential oil constituents from plants are used as alternative treatments for a wide range of illnesses including cancer prevention and treatment [7]–[9]. Recently, studies have reported that essential oils, including the leaf of *Porcelia macrocarpa* [10] and *Pyrolae herba* [11], the heartwood of *Cunninghamia lanceolata* var. konishii [12], and the seed of *Litsea cubeba* [13], possess anticancer effects against different types of human tumors. Taiwan is located in the subtropics and has abundant plants with essential oil extracts that have been suggested as potential candidates for new therapeutic compounds. Many essential oils are volatile, which may represent an advantage for lung cancer treatment; this consideration prompted us to screen the potency of essential oils against lung adenocarcinomas [13].

In this study, we screened over 40 different essential oils from 31 different indigenous plants from Taiwan to identify new strategies for overcoming treatment failure. Among the investigated substances, we determined that hinokitiol, from the essential oil of *Calocedrus formosana* heartwood, had the most potent anticancer effects on EGFR-TKI-resistant lung cancer cell lines (H1975 and PC9-IR). Here, we reveal the novel mechanisms by which hinokitiol exerts its potent anticancer effects on several lung adenocarcinoma cell lines as well as EGFR-TKI-resistant cells. Specifically, hinokitiol induces DNA damage, autophagy, cell cycle arrest in S phase, and senescence. The potential anti-tumor effect and mechanisms of hinokitiol were confirmed in a xenograft model. Our findings suggest that hinokitiol could be a promising compound for treating EGFR-TKI-resistant lung adenocarcinomas.

## Materials and Methods

### Essential oils and chemicals

A total of 40 essential oils from 31 local plants in Taiwan were extracted using a standard hydrodistillation technique, and the constituents were analyzed through GC-MS. Hinokitiol (β-thujaplicin) was purchased from Sigma (St. Louis, MO, USA) and dissolved in DMSO as a stock stored at −20°C. 3-methyladenine (3-MA) was purchased from Sigma (M9281) and dissolved in RPMI complete medium (Gibco, Breda, The Netherlands). Chloroquine was purchased from Sigma (C6628) and dissolved in DMSO as a stock stored at −20°C. Acridine orange was purchased from Sigma (A6014).

### Cell lines and culture conditions

The human lung adenocarcinoma cell lines, A549 (EGFR wild type), H1975 (EGFR L858R/T790M, gefitinib-resistant), H1299 (EGFR wild type, p53 null), and H3255 (EGFR L858R) were purchased from American Type Culture Collection (ATCC; Manassas, VA, USA). PC9 (EGFR exon 19 deletion) and PC9-IR (EGFR exon 19 deletion, gefitinib-resistant) were kind gifts from Dr. C. H. Yang (Graduate Institute of Oncology, Cancer Research Center, National Taiwan University). Human stromal fibroblast tissues were harvested from freshly resected lung tumor tissues from lung cancer patients who underwent surgical resection at the National Taiwan University Hospital and were sampled at least 5 cm away from neoplastic lesions by a pathologist within 30 min. The detail processes and protocols of isolating human stromal fibroblasts were described as our previous report [14]. This research project was approved by the institutional review board of National Taiwan University College of Medicine (Taipei, Taiwan) and written informed consent was obtained from all patients. The cell lines including stromal fibroblasts were cultured in RPMI-1640 medium supplemented with 10% fetal bovine albumin and 1% penicillin/streptomycin in a humidified atmosphere of 5% CO_2_ in air at 37°C.

### Cell proliferation assay

The effects of essential oils on A549 cells were evaluated using MTT (3-(4,5-dimethylthiazol-2-yl)-2,5-diphenyltetrazolium bromide) assay. The effects of hinokitiol on a series of lung adenocarcinoma cell lines were assayed through trypan blue staining. For the MTT assay, 5×10^3^ cells were cultured in 96-well plates overnight and then incubated with the essential oils under investigation (diluted 1∶10,000 in medium) for 48 h. At the indicated times, the medium was removed, and 0.5 mg/ml MTT solution, which was dissolved in the culture medium, was added to the wells. After a further 1.5 h of incubation, the medium was removed, and DMSO was added to the plates. The color intensity was measured at 570 nm using a multi-label plate reader (Vector3; Perkin-Elmer, USA). For trypan blue staining, 2×10^4^ cells were cultured in 12-well plates overnight and then incubated with 0.3125–10 µM hinokitiol for 24, 48, and 72 h. At the indicated times, the cells were trypsinized and stained with trypan blue. The viable cells that excluded trypan blue were counted in a counting chamber. For the 3-MA treated experiment, 5.5×10^3^ cells were cultured in 96-well plates overnight and then incubated with 2.5 mM 3-MA for 1 hour prior to 5 µM hinokitiol treatment for 48 h. At the indicated times, the cells were trypsinized and stained with trypan blue. The viable cells were counted in a counting chamber.

### Colony formation assay

H1975 and PC9-IR cells were cultured overnight in a 6-well plate at a density of 80 cells per well. Hinokitiol was freshly prepared at concentrations of 0.5, 1, or 5 µM and added to the wells. The cells were then incubated for 3 days. On the 4^th^ day, the cells were incubated with drug-free complete medium and cultured for another 7–10 days. The colonies were fixed in 4% ice-cold paraformaldehyde for 15 minutes at 37°C, and each well was stained with 0.1% crystal violet overnight at room temperature. The colonies were then counted.

### Gene expression profile by Affymetrix array analysis

The microarray experiments were performed using the Affymetrix Human Genome GeneChip expression by the Microarray Core Facility of National Taiwan University according to the manufacturer's protocols (Affymetrix, Santa Clara, CA, USA). All experiments were performed with complementary RNA probes prepared from H1975 or PC9-IR cells treated with or without 5 µM hinokitiol. All data were collected and analyzed according to the Affymetrix manual, and the raw microarray data were uploaded to the CRSD2 web server made in-house [15] and to GeneGo (http://www.genego.com/metacore) for pathway analysis.

### DNA damage and autophagy gene expression profiling using real-time PCR arrays

Human DNA damage and autophagy signaling PCR arrays (SuperArray Bioscience), each of which assessed the expression of 84 genes, were used to assess the effect of hinokitiol on H1975 cells and stromal fibroblasts. The synthesis of complementary DNA, real-time PCR, and statistical analyses were performed according to the manufacturer's instructions, and the data shown represent the average of three replicates.

### Cell cycle analysis

For propidium iodide (PI; Sigma) staining, H1975 cells and lung stromal fibroblasts (5×10^5^) were cultured in 6-cm dishes overnight and then incubated with 5 µM hinokitiol for 72 h. At the indicated times, the cells were trypsinized and fixed in 70% ice-cold alcohol at −20°C overnight. The fixed cells were washed twice in phosphate-buffered saline (PBS) and resuspended in a solution containing 0.1% Triton X-100, 200 µg/ml RNase A, and 20 µg/ml PI in PBS. The cells were incubated for 20 minutes at room temperature in the dark, and the stained nuclei were analyzed using a flow cytometer (FC500, Beckman Coulter). 5-Bromodeoxyuridine (BrdU)-labeled cells were measured using a BrdU flow kit (BD Pharmingen, USA) according to the manufacturer's instructions. Briefly, H1975 cells (5×10^5^) were cultured in 6-cm dishes overnight and then incubated with 5 µM hinokitiol for 72 h. The cells were labeled with 10 µM BrdU for 12 h before harvesting. At the indicated times, the BrdU-labeled cells were fixed, permeabilized, stained and analyzed using a flow cytometer (FC500, Beckman Coulter).

### Annexin V staining

Apoptosis was measured using an annexin V apoptosis assay kit (BD Pharmingen, USA) according to the manufacturer's instructions. Briefly, H1975 cells and lung stromal fibroblasts (5×10^5^ cells) were cultured in 6-cm dishes overnight and then incubated with 5 µM hinokitiol for 72 h. At the indicated times, both floating and attached cells were collected and stained with annexin V-conjugated with Alexa Fluor 488 dye and PI. The stained cells were analyzed using a flow cytometer (FC500, Beckman Coulter).

### Acridine orange staining

To assess the formation of acidic vesicular organelles (AVOs), H1975 cells (5×10^5^ cells) were seeded in 6-cm dishes with 5 µM hinokitiol for 8 h. At the indicated times, the cells were washed with PBS twice, followed by staining with 1 µg/ml acridine orange (Sigma, A 6014) in PBS containing 5% fetal bovine serum (FBS) at 37°C for 15 min. The stained cells were harvested and washed twice with PBS. The stained cells were then resuspended in PBS with 5% FBS and analyzed using a flow cytometer (FC500, Beckman Coulter).

### Western blot analysis

H1975, H1299, and lung stromal fibroblasts (5×10^5^) were cultured in 6-cm dishes overnight and then incubated with 5–25 µM hinokitiol for 4–72 h. Whole cell lysates were prepared using mammalian protein extraction reagent (Pierce, Rockford) containing protease inhibitors (Cytoskeleton, PICO2) and phosphatase inhibitors (Sigma). The lysates were clarified by centrifugation. Each sample, containing 40 µg protein, was separated on a 7.5–15% sodium dodecyl sulfate polyacrylamide gel (SDS–PAGE). The proteins were transferred by electroblotting to nitrocellulose membranes. The membranes were blocked for 60 min in 5% skim milk in TBST (Tris-buffered saline containing 20 mM NaF, 2 mM EDTA, and 0.2% Tween 20) at room temperature. Immunoblotting was performed using the following specific primary antibodies: total EGFR (Cell Signaling Technology, #2232S), phospho-EGFR (Y1068, Cell Signaling Technology, #2236S), and total ERK (Zymed Laboratories, 13-6200); phospho-ERK (T202/Y204, BD Transduction Laboratories, 612358); p21 (Santa Cruz C-19); cyclin D1 (Santa Cruz A-20); cyclin E2 (Cell Signaling Technology, #4132); cyclin A2 (GeneTex GTX103042); cyclin B1 (GeneTex GTX100911); γ-H2AX (Ser139, Millipore, #05-636); LC3 (Cell Signaling Technology, #3868); total ATM (GeneTex GTX70103); phospho-ATM (Ser1981, GeneTex, GTX61739); total SMC3 (Bethyl A302-068A); phospho-SMC3 (Ser1981, Bethyl, A300-480A); total p53 (Santa Cruz DO-1); phospho-p53 (Ser 15, GeneTex, GTX21431); p62 (Cell Signaling Technology, #5114S) and ATG5 (Cell Signaling Technology, #12994). The secondary antibodies were horseradish peroxidase-conjugated immunoglobulins. The antibodies were used according to the conditions recommended by the manufacturers. The bound antibodies were detected using the Enhanced Chemiluminescence System (Santa Cruz). Chemiluminescent signals were captured using the Fujifilm LAS 3000 system (Fujifilm, Tokyo, Japan). The expression level of each protein was quantified with the NIH ImageJ program using β-actin as a loading control.

### Immunofluorescence γ-H2AX focus assay and confocal microscopy

H1975 cells were seeded on coverslips, allowed to attach overnight, and incubated with 5 µM hinokitiol for 48 h. After hinokitiol treatment, the cells were fixed with 4% paraformaldehyde for 10 min at room temperature and washed three times with PBS. After fixation, the cells were permeabilized with 0.5% Triton X-100 in PBS for 5 min and then blocked with 0.1% Tween 20 and 1% BSA in PBS for 5 min. The cells were incubated for 1 h with a mouse anti-γ-H2AX monoclonal antibody (Upstate, 1∶800 dilution) and stained with DAPI (Sigma, D9542) and phalloidin dye (Alexa flour 647). The stained cells were examined using a confocal laser scanning microscope (TCS SP5, Leica) at 400× magnification. All experiments were performed in triplicate.

### β-Galactosidase staining for senescence

Senescence-associated-β-gal (SA-β-Gal) activity was measured using a β-gal staining kit (Cell Signaling Technology, #9860) at pH 6 according to the manufacturer's instructions. Briefly, 1×10^5^ cells were incubated with 5 µM hinokitiol for 72 h, washed once with PBS, and fixed with 2% formaldehyde and 0.2% glutaraldehyde in PBS for 15 min. The cells were then washed twice with PBS and stained with a solution containing 5-bromo-4-chloro-3-indolyl-b-D-galactopyranoside (X-gal). Following incubation for 10–12 h at 37°C, senescent cells were identified by their blue staining using standard light microscopy (DMIRB, Leica).

### siRNA transfection

Knockdown using specific RNA and scramble RNA interference was performed. Briefly, a mixture of 2 µg ATG5 siRNA plasmid (Invivogen, psirna42-hatg5) or scramble siRNA plasmid (Invivogen, ksirna42-lucgl3) and transfection reagent (Lipofectamine, Invitrogen) in serum-free culture medium was incubated with H1975 cells (2.5×10^5^ cells) in 6-well dishes for 6 h. Then, complete culture medium and 5 µM hinokitiol were added, and the samples were incubated for 72 h. Corresponding protein down-regulation was confirmed using western blot analysis.

### Ethics statement

All animal manipulations were performed in the Laboratory Animal Center of National Taiwan University College of Medicine (Taipei, Taiwan) in accordance with the protocols approved by the Institutional Animal Care and Use Committee of National Taiwan University College of Medicine (Taipei, Taiwan). All manipulations were performed humanely under isoflurane anesthesia, and all efforts were made to minimize suffering.

### Animal model and experimental protocol

Six-week old NOD-SCID mice were purchased from LASCO Charles River Technology (Taiwan) and maintained in a specific pathogen-free environment. For subcutaneous xenografts, H1975 cells (1×10^6^ cells in 100 µl of HBSS) were injected subcutaneously in the right flank of the animals. The hinokitiol treatment (2 or 10 mg/kg in PBS with 5% DMSO, i.p.) was initiated when the tumors reached 20 mm^3^ and was administered daily until the animals were sacrificed at days 14 or 21. The tumors were measured every 4 days using a caliper, and the tumor volume was calculated according to the following formula: V = 0.4×*a^2^b*, where *a* refers to the smaller diameter and *b* is the diameter perpendicular to *a*. At the end of the experiments, the mice were humanely euthanized through CO_2_ inhalation to minimize suffering. Tumor xenografts were removed and weighed. A sample of tissue from each tumor was fixed in formalin and then embedded in paraffin.

### Histological and immunohistochemical staining analysis

Paraffin sections were deparaffinized with xylene and then submitted to hematoxylin and eosin staining and immunohistochemical staining. Hematoxylin and eosin staining was carried out using standard techniques. For the immunohistochemical staining, the paraffin sections were deparaffinized with xylene, and the antigens were retrieved by incubation in 0.01 M, pH 6.0 citrate buffer at 95°C for 20 min. The slides were then incubated in blocking buffer (3% BSA and 0.2% triton x-100 in PBS) for 1 h at room temperature. The primary antibodies (γ-H2AX, Ser139, Millipore, #MABE205; LC3, Cell Signaling Technology, #3868) were applied overnight at 4°C, and then washed three times with PBS for 5 min. These antibodies were detected using the IHC Select HRP/DAB kit (#DAB150, Millipore) according to the manufacturer's instructions. The slides were incubated for 1 h with biotinylated secondary antibody at room temperature, and then washed three times with PBS for 5 min. The slides were incubated for 30 min with streptavidin-HRP at room temperature, and then washed three times with PBS for 5 min. The substrate was developed using 4% DAB and the sections were counterstained with hematoxylin. The sections were dehydrated through graded alcohols, immersed in xyline, and mounted with coverslips. The tissue sections were observed under a standard light microscope (BX51, Olympus).

### Statistical Analyses

All experiments were performed in triplicate and analyzed using the t-test (Excel; Microsoft) for significant differences. P values of <0.05 were considered significant.

## Results

### Screening for essential oils with anti-proliferative effects on lung adenocarcinoma cells

The essential oils isolated from 40 indigenous plants were evaluated for anti-proliferative effects in the human lung cancer cell line, A549, after 48 h of treatment. As shown in Table 1, five potent essential oils from *Calocedrus formosana* heartwood, *Machilus japonica* Sieb. and Zucc, *Eucalyptus camaldulensis* leaf, *Nothaphoebe konishii* (*Hay*.), and *Cunninghamia konishii* heartwood reduced cell proliferation to 64.7±6.5%, 69.9±1.8%, 67.1±6.7%, 67.7±1.7%, and 71.4±3.4% of control cells, respectively. The most potent essential oil, *Calocedrus formosana* heartwood extract, was selected for further evaluation.

**Table 1:** The screening of essential oils on A549 cell proliferation as determined using MTT assay.

Essential oils	Proliferation rate (%)
*Eucalyptus camaldulensis* flower	93.2±13.2
*Eucalyptus grandis* leaf	95.8±8.7
*Cinnamomum subavenium Miq*.(Fu-Shan No. 4)	101.6±8.6
*Cunninghamia konishii* leaf	100.4±1.0
*Eucalyptus camaldulensis* leaf	97.8±4.6
*Cinnamomum subavenium Miq.* (Dasyue Mountain)	95.8±1.6
*Cinnamomum subavenium Miq* (Liouguei)	92.6±8.7
*Eucalyptus urophylla* leaf	100.1±2.5
*Cinnamomum camphora (L.) Presl* twig	101.3±2.8
*Cinnamomum camphora (L.) Presl* bark	99.7±0.1
*Cinnamomum camphora (L.) Presl* flower	102.1±4.1
*Cinnamomum camphora (L.) Presl* heartwood	97.8±2.7
*Litsea cubeba* stem	98.0±2.4
*Litsea cubeba* fruit	88.9±8.9
*Litsea cubeba* old twig	87.4±0.4
*Litsea cubeba* leaf	104.1±1.4
*Litsea cubeba* young twig	100.7±1.6
*Cryptomeria japonica* heartwood	88.8±2.6
*Eucalyptus urophylla* flower	107.8±3.5
*Taiwania cryptomerioides* Hayata leaf	87.4±0.7
*Calocedrus Formosana* heartwood	64.7±6.5*
*Calocedrus formosana* leaf	93.9±3.5
*Taiwania cryptomerioides* Hay. leaf	89.4±2.7
*Houttuynia cordata* Thunb	95.7±6.1
*Machilus japonica* Sieb. & Zucc (Chihtuan No. 1)^#^	69.9±1.8*
*Machilus japonica* Sieb. & Zucc (Chihtuan No. 2)^#^	100.1±9.3

Essential oils	Proliferation rates (%)
*Eucalyptus camaldulensis* leaf	67.1±6.7*
*Nothaphoebe konishii (Hay.)*	67.7±1.7*
*Machilus japonica* Siebold et Zucc^#^	101.1±0.8
*Cunninghamia konishii* heartwood	71.4±3.4*
*Cinnamomum camphora (L.)* Presl leaf	100.2±0.2
*Chamaecyparis formosensis* bark	98.2±1.9
*Chamaecyparis formosensis* sapwood	95.8±7.4
*Machilus thunbergii*	92.6±4.5
*Beilschmiedia erythrophloia* Hayata	97.9±0.6
*Clausena excavata* Burm. f.	100.0±1.7
*Juniperus formosana* Hayata leaf	98.8±3.9
*Lavandula pinnata*	100.5±2.7
*Nepeta cataria L.*	101.0±6.2

The proliferation rates (%) are represented as the mean ± SD (%) compared with the corresponding control (0.1% DMSO) at 48 h (*n = *3). * indicates the top 5 potent essential oils. ^#^ belongs to the same species but was collected in different geographic areas in Taiwan.

### Hinokitiol inhibits the proliferation of gefitinib-resistant human lung cancer cell lines

**Figure 1:**
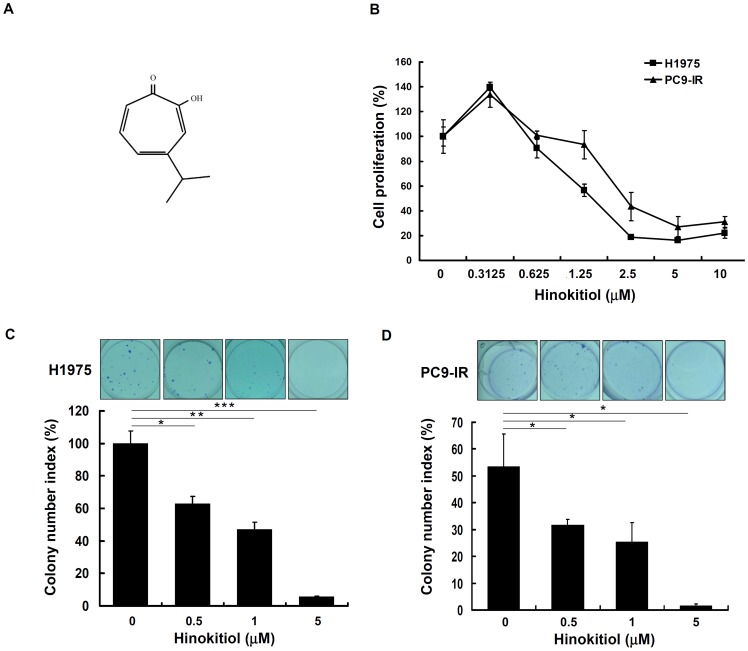
The effects of hinokitiol on cell proliferation. (A) The chemical structure of hinokitiol. (B) The effect of a 72-h hinokitiol treatment on H1975 and PC9-IR cell proliferation, as assayed through trypan blue staining. (C) The effect of hinokitiol on the colony formation ability of H1975 cells. (D) The effect of hinokitiol on the colony formation ability of PC9-IR cells. In (B), (C), and (D), the results are representative of three different experiments and are expressed as the mean ± SD and as % of control. *, **, and *** indicate a significant difference at the level of *p*<0.05, *p*<0.01, and *p*<0.001, respectively.

Hinokitiol is the major active compound in the essential oil of *Calocedrus formosana* heart wood [16], and its chemical structure is shown in Figure 1A. To investigate the potential anticancer activity of hinokitiol on human lung adenocarcinoma cells, six different human lung adenocarcinoma cell lines with different EGFR status, A549 (EGFR^wt^), PC9 (EGFR^del19^), H1299 (EGFR^wt^), H3255 (EGFR^L858R^), PC9-IR (EGFR^del19^, with resistance to gefitinib) and H1975 (EGFR^L858R+T790M^, with resistance to gefitinib) cells, were treated with hinokitiol (5 and 10 µM) for 48 and 72 h. Then, cell proliferation was evaluated by directly counting cells after trypan blue staining. As shown in Table 2, hinokitiol inhibited the proliferation of all cells in a time- and concentration-dependent manner. Interestingly, the gefitinib-resistant cell lines, H1975 and PC9-IR, were inhibited by hinokitiol at a dose similar to that required for the gefitinib-sensitive cell lines PC9 and H3255. We further focused on the effects and underlying mechanisms of the action of hinokitiol on the gefitinib-resistant cells, H1975 and PC9-IR [17], [18]. We found that hinokitiol had IC_50_ values of 1.57 and 1.87 µM (72 h) in H1975 and PC9-IR cells, respectively (Fig. 1B). In addition, Figure 1C and 1D show that hinokitiol inhibited the colony formation ability of H1975 and PC9-IR cells in a concentration-dependent manner with an IC_50_ <1 µM. These results indicated that hinokitiol potently reduced the proliferation and colony formation potential of H1975 and PC9-IR cells.

**Table 2:** The effect of hinokitiol on adenocarcinoma cell proliferation as determined through trypan blue staining.

Hinokitiol	48 h			72 h		
Conc. (µM)	0	5	10	0	5	10
A549	100±4.0	52.7±3.6	34.7±5.2	100±7.7	28.9±1.1	18.2±7.2
PC9	100±21.2	52.8±7.8	22.0±8.9	100±7.9	21.9±4.2	15.9±1.8
H1299	100±24.4	82.8±0	41.4±19.5	100±20.2	12.7±4.5	15.1±1.1
H3255	100±18.7	37.0±4.3	22.6±16.0	100±4.9	25.3±3.3	18.4±6.5
PC9-IR	100±12.5	73.5±12.5	36.8±6.2	100±1.1	37.4±3.2	8.4±3.2
H1975	100±9.4	55.3±3.4	40.3±10.8	100±8.1	38.9±12.5	16.8±6.5

The number of viable cells was determined through trypan blue staining, and the proliferation rates (%) are represented as the mean ± SD (%) compared with the corresponding control (0.1% DMSO) at the indicated times (*n = *3).

### Transcriptomic and pathway analyses showing the potential molecular mechanisms of the effects of hinokitiol on H1975 and PC9-IR cells

**Figure 2:**
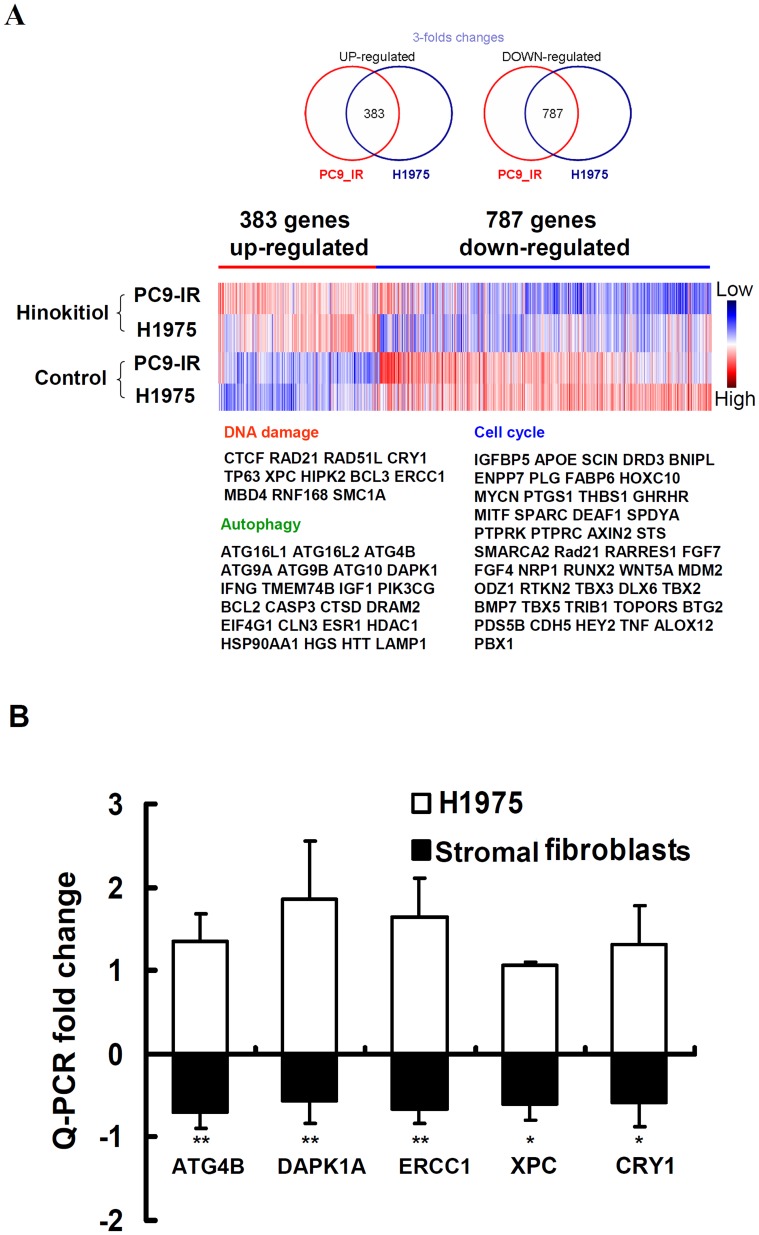
The effects of hinokitiol on gene expression. **(A)** Microarray profiling of H1975 cells and PC9-IR cells treated with 5 µM hinokitiol for 48 h. **(B)** Q-PCR array validation of the expression of genes related to DNA damage and autophagy in H1975 cells and lung stromal fibroblasts after 5 µM hinokitiol treatment for 24 h. The results are representative of those obtained in three different experiments and are expressed as the fold change compared with control. * and ** indicate a significant difference at the level of *p*<0.05 and *p*<0.01, respectively.

To study the potential mechanisms of hinokitiol on gefitinib-resistant lung adenocarcinoma cells, we compared the gene expression profiles of H1975 and PC9-IR cells with or without hinokitiol using the Affymetrix human GeneChip. Here, we found that 383 genes were up-regulated over 3 fold, and 787 genes were down-regulated over 3 fold in both cell lines after 5 µM hinokitiol treatment for 48 h (Fig. 2A). The CRSD2 web server, Gene Ontology and Pathway Enrichment analysis, predicted that hinokitiol could affect certain key regulators/factors involved in DNA damage, autophagy, and cell cycle signaling in both cell lines. Furthermore, we examined DNA damage- and autophagy-related genes in H1975 cells and human lung stromal fibroblasts upon hinokitiol treatment using Q-PCR array (SuperArray Bioscience). We confirmed that two autophagy-related genes, *ATG4B* and *DAPK1A*, were up-regulated by hinokitiol treatment in H1975 cells but were down-regulated in stromal fibroblasts (>1.5-fold change; Fig. 2B). In addition, three DNA damage-related genes, *ERCC1*, *XPC*, and *CRY1*, were up-regulated in hinokitiol-treated H1975 cells but down-regulated in stromal fibroblasts (>1.5-fold change). These results indicated that hinokitiol induced the expression of certain DNA damage- and autophagy-related genes in cancer cells but not in human stromal fibroblasts.

### Hinokitiol caused DNA damage in a p53-independent manner in lung adenocarcinoma cells but not in human lung stromal fibroblasts

**Figure 3:**
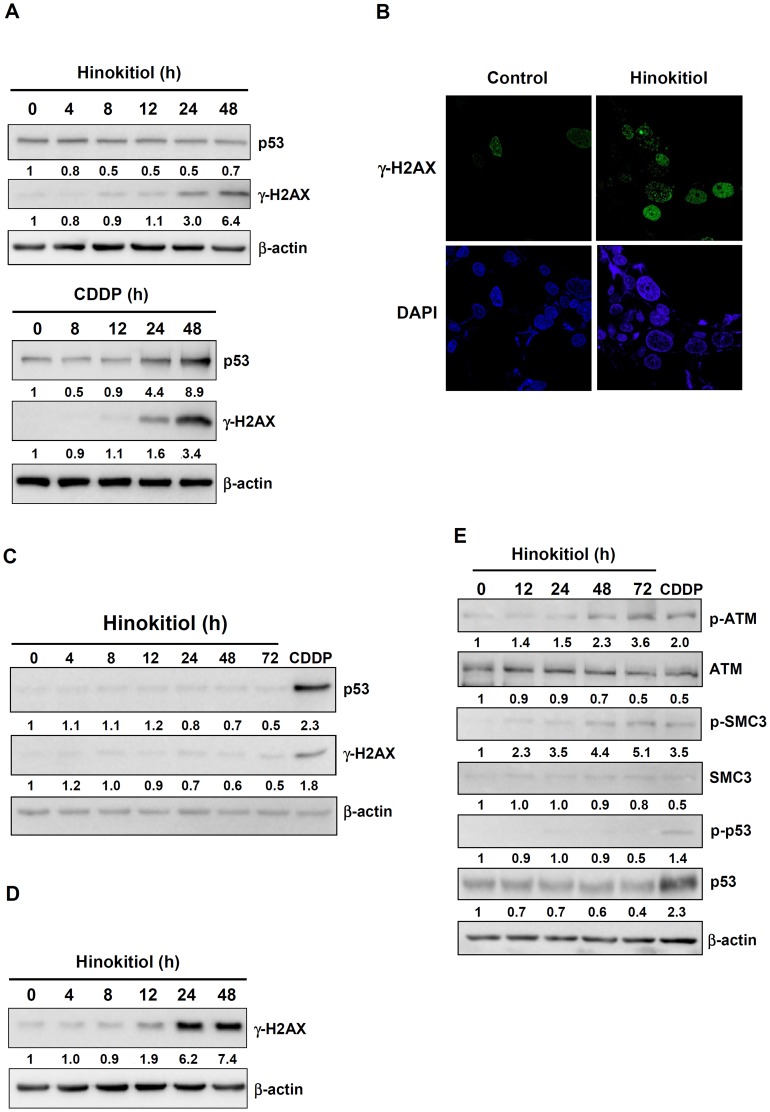
The effects of hinokitiol on the expression of DNA damage regulatory proteins. **(A)** The effect of hinokitiol (5 µM) or cisplatin (25 µM) on the level of γ-H2AX phosphorylation and total p53 expression in H1975 cells, as assayed using western blots. **(B)** Assessment of hinokitiol-induced DNA damage in H1975 cells through an immunofluorescence γ-H2AX focus assay. **(C)** The effect of hinokitiol (5 µM) on the level of γ-H2AX phosphorylation and total p53 expression in lung stromal fibroblasts, as assayed using western blots. **(D)** The effect of hinokitiol (5 µM) on the level of γ-H2AX phosphorylation in H1299 cells. **(E)** The effect of hinokitiol (25 µM) or cisplatin (CDDP, 25 µM) on the phosphorylation and total level of ATM, SMC3, and p53 in H1975 cells. The expression level of each protein was quantified with the NIH ImageJ program using β-actin as a loading control.

According to our genome-wide transcriptomic analysis and Q-PCR validation, we found that the DNA damage-related genes *ERCC1*, *XPC*, and *CRY1* were up-regulated in hinokitiol-treated lung cancer cells. To further investigate whether hinokitiol can cause DNA damage, the levels of phosphorylated γ-H2AX and total and phosphorylated p53 were examined. Figure 3A shows that the levels of phosphorylated γ-H2AX were augmented after 48 h of hinokitiol treatment in H1975 cells, whereas total p53 was unchanged (Fig. 3A). The effect of hinokitiol on γ-H2AX phosphorylation was confirmed by immunostaining, which showed γ-H2AX protein accumulation in the nucleus of H1975 cells treated with hinokitiol (Fig. 3B), indicating that hinokitiol induced DNA damage in H1975 cells. Interestingly, hinokitiol did not induce DNA damage in human lung stromal fibroblasts (Fig. 3C), and this result correlated with the expression of genes related to DNA damage shown in Figure 2. To confirm whether hinokitiol-induced DNA damage occurred independent of p53, we treated p53-null H1299 cells with hinokitiol and found that hinokitiol still induced DNA damage in these cells (Fig. 3D). Furthermore, we detected the major regulatory pathway of DNA damage response in the H1975 cells, such as the levels of phosphorylated and total ATM and SMC3. Additionally, we further detected the phosphorylated p53 to corroborate the DNA damage response is independent of p53 status evidenced by the phosphorylated or total p53 were unchanged by hinokitiol treatment (25 µM hinokitiol; Fig. 3E).

### Hinokitiol induced autophagy in lung adenocarcinoma cells but not in human lung stromal fibroblasts

**Figure 4:**
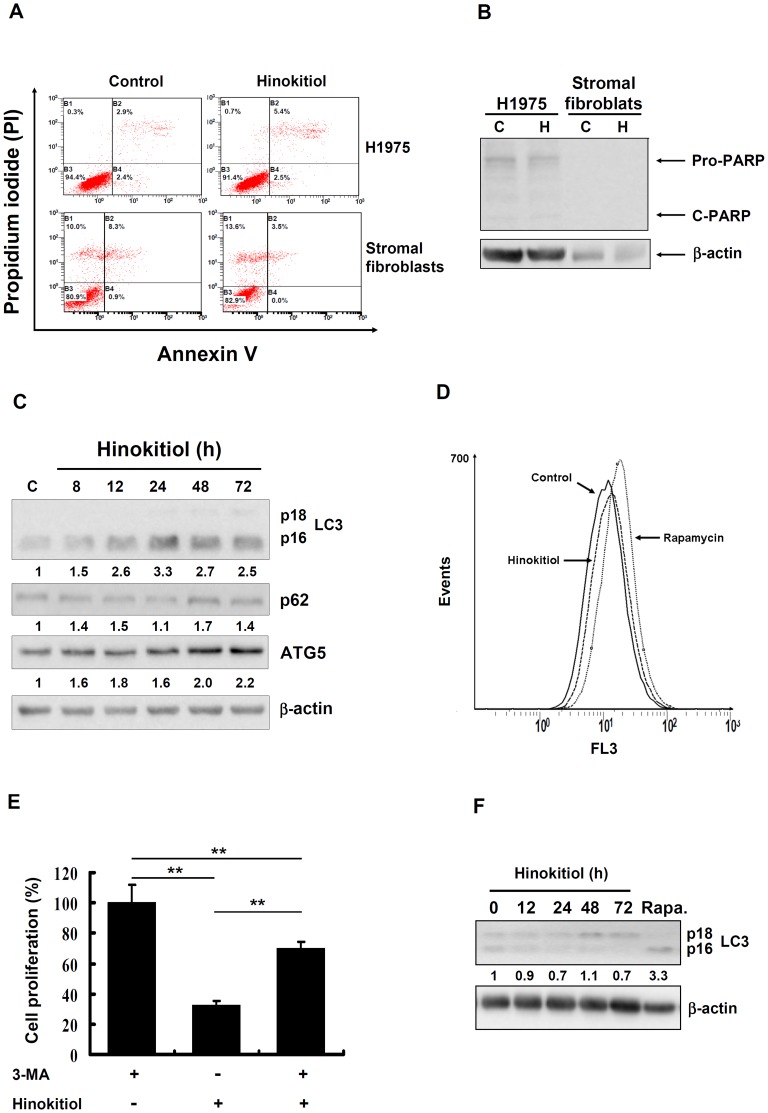
The effects of hinokitiol on apoptosis and autophagy. (**A**) Apoptosis was assessed using an annexin-V/PI binding assay in H1975 cells and lung stromal fibroblasts after 5 µM hinokitiol treatment. Western blot analysis of PARP in H1975 cells and lung stromal fibroblasts (**B**), LC3, p62 and ATG5 expression in (**C**) H1975 cells and (**F**) lung stromal fibroblasts. The treatment of 100 nM rapamycin for 48 h was used as a positive control for LC3 expression. The expression level of each protein was quantified with the NIH ImageJ program using β-actin as a loading control. (**D**) The formation of AVOs was quantified by flow-cytometry after acridine orange staining in H1975 cells treated with 5 µM hinokitiol for 8 h. (**E**) H1975 cells were pretreated with 2.5 mM of 3-MA for 1 h, followed by exposure to 5 µM hinokitiol for 48 h. Cell proliferation was analyzed through a trypan blue staining assay. The results are representative of three different experiments and are expressed as the mean ± SD. ** indicates a significant difference at the level of *p*<0.01.

To gain further insight into the mode of action by which hinokitiol limited cancer cell proliferation, the effect of hinokitiol on apoptosis was examined by flow cytometry with annexin V-FITC/PI staining in H1975 cells. We found that hinokitiol treatment for 72 h did not significantly affect the percentage of cells in early or late apoptosis (Fig. 4A). Hinokitiol also did not induce apoptosis in human stromal fibroblasts (Fig. 4A). In addition, hinokitiol treatment did not induce detectable PARP cleavage in H1975 cells or human stromal fibroblasts (Fig. 4B). These results prompted us to investigate whether hinokitiol induced autophagy in H1975 cells. We found that the expression of LC3-II, p62 and ATG5 proteins, which are markers of autophagosome formation [19], [20], increased after the hinokitiol treatment (Fig. 4C). Figure 4E provides additional evidence that hinokitiol induces cell autophagy, showing that 3-MA, an autophagy inhibitor, partially rescued the inhibition of cell growth induced by hinokitiol. In addition, we confirmed the autophagic response to hinokitiol by the analysis of the formation of AVOs. The flow cytometry analysis showed that the number of acidic vesicles in the H1975 cells slightly increased after hinokitiol exposure (Fig. 4D). Interestingly, hinokitiol did not induce significant levels of autophagy in human stromal fibroblasts (Fig. 4F), and this result correlated with the expression of genes related to autophagy shown in Figure 2.

### Effects of hinokitiol on cell cycle arrest

**Figure 5:**
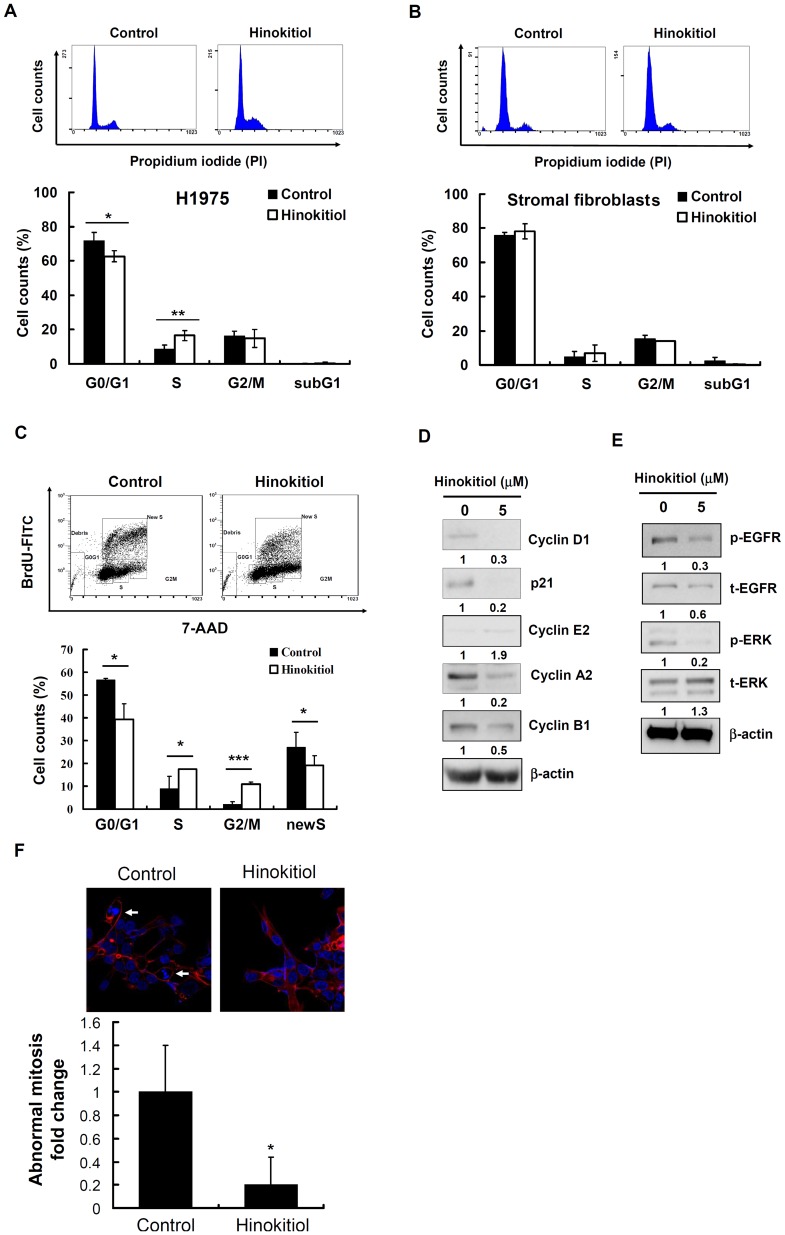
The effect of hinokitiol on cell cycle distribution. H1975 cells **(A)** and lung stromal fibroblasts **(B)** were treated with 5 µM hinokitiol for 72 h. The cell cycle distribution was determined by flow cytometry after the nuclei were stained with PI. **(C)** BrdU incorporation assay was applied in H1975 cells treated with 5 µM hinokitiol for 72 h. **(D)** Western blot analysis of cyclin D1, p21, cyclin E2, cyclin A2, and cyclin B1 expression in H1975 cells. **(E)** Western blot analysis of EGFR and ERK expression in H1975 cells. The expression level of each protein was quantified with the NIH ImageJ program using β-actin as a loading control. **(F)** Abnormal mitotic morphology stained with DAPI and phalloidin were quantified at 400× magnification under a confocal microscope (TCS SP5, Leica). In **(A)**, **(B)** and (**C**), the results are representative of three different experiments, and the histogram shows the quantification expressed as the mean ± SD. *, ** and *** indicate a significant difference at the level of *p*<0.05, *p*<0.01 and <0.001, respectively. In **(F)**, the histogram shows the quantification expressed as the mean ± SD of ratio in 5-10 fields per coverslip. * indicates significant differences at the level of *p*<0.05.

We observed that hinokitiol reduced the proliferation of cancer cells, but this was not due to cytotoxicity (Fig. 4A & B). As such, we examined the effect of hinokitiol treatment on the cell cycle distribution of H1975 cells and found that the ratio of cells in S phase significantly increased after hinokitiol treatment. Concomitantly, the percentage of cells in the G1 phase was reduced compared with control cells. This result indicated that hinokitiol induced the accumulation of cancer cells in the S phase of the cell cycle (Fig. 5A). Interestingly, this effect on cell cycle distribution was not significantly observed in human lung stromal fibroblasts treated with hinokitiol (Fig. 5B). Furthermore, we used the BrdU flow assay to corroborate the S-phase arrest data in response to hinokitiol exposure in H1975 cells. In Fig. 5C, the percentage of BrdU-negative cells in S-phase was higher in the hinokitiol exposure group; whereas the newly incorporated BrdU-labeled cells in S-phase were lower in H1975 cells. Moreover, both cancer and stromal fibroblasts in the sub-G1 phase were unaffected by the treatment with hinokitiol (Fig. 5A & B); these results were associated with the lack of apoptosis in H1975 cells, as shown in Fig. 4A & B. To investigate the underlying mechanism by which hinokitiol treatment induced cell-cycle arrest at S phase, we examined the key regulators during cell cycle progression. We found that the protein levels of cyclin D1, p21, cyclin A2, and cyclin B1 were down-regulated and that the levels of cyclin E2 were 1.9 times up-regulated in response to a 72-h treatment with hinokitiol compared with control (Fig. 5D). In addition, we found that the phosphorylation levels of EGFR and ERK, the up-stream signaling regulators of cyclin D1 [21], were significantly reduced after long-term treatment with hinokitiol (5 µM hinokitiol, 72 h; Fig. 5E). The nuclear staining in H1975 cells revealed that the proportion of abnormal mitosis was reduced after 5 µM hinokitiol exposure for 72 h (Fig. 5F).

### Hinokitiol induced cellular senescence in both human lung cancer cells and lung stromal fibroblasts

**Figure 6:**
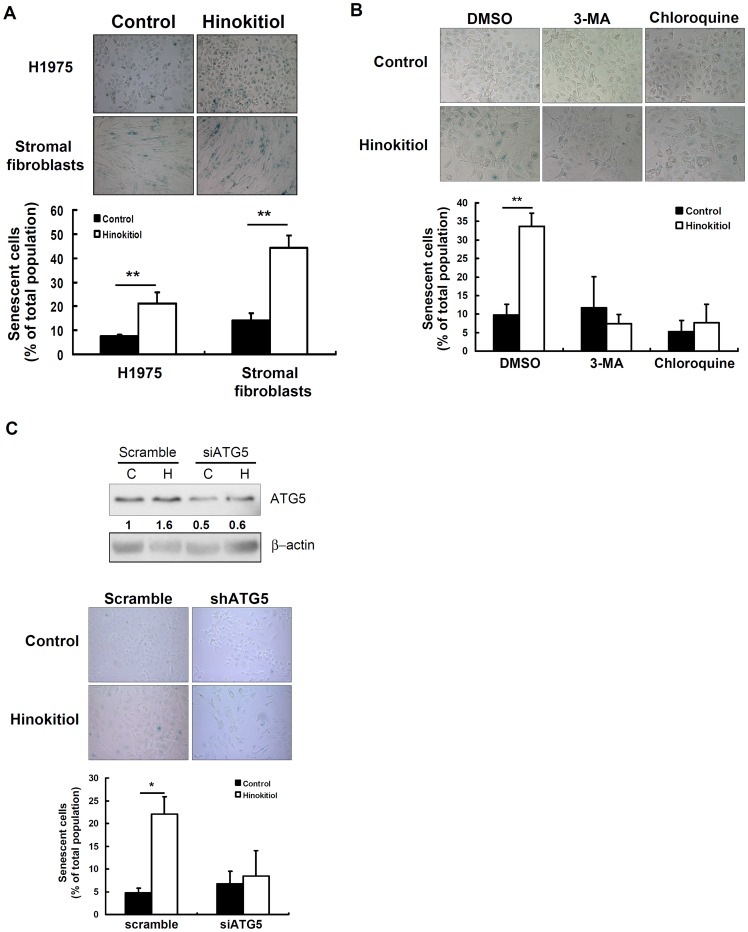
Hinokitiol induced cellular senescence in H1975 cells and lung stromal fibroblasts. **(A)** The senescent cells were quantified at 200× magnification under a standard light microscope. **(B)** Hinokitiol induced cellular senescence was attenuated by autophagy inhibitors in H1975 cells. **(C)** Hinokitiol induced cellular senescence was attenuated by transfection of siRNA against ATG5 in H1975 cells. Corresponding protein expression was detected by western blot. The expression level of each protein was quantified with the NIH ImageJ program using β-actin as a loading control. In **(A)**, **(B)** and **(C)**, each value is the mean ± SD of 3-5 fields of three different experiments. * and ** indicate a significant difference at the level of *p*<0.05 and *p*<0.01, respectively.

Taken together, our results showed that hinokitiol inhibited cell proliferation by inducing DNA damage, autophagy, and cell cycle arrest in lung adenocarcinoma cells but not in human lung stromal fibroblasts. Because apoptosis and autophagy were not observed in hinokitiol-treated fibroblasts, we sought to evaluate whether cellular senescence could be triggered by hinokitiol treatment. The effect of hinokitiol on cellular senescence was assessed through SA-β-Gal staining, and we found that hinokitiol treatment (5 µM, 72 h) induced cellular senescence in H1975 cells and, more significantly, in human lung stromal fibroblasts (Fig. 6A). Next, we further clarify whether autophagy induced senescence in the H1975 cells after hinokitiol treatment. Thus, we used the autophagy inhibitors 3-MA and chloroquine and transfected the cells with siRNA against ATG5 to evaluate the hinokitiol-induced senescence. In Figure 6B & C, hinokitiol-induced senescence was attenuated by cotreatment with 3-MA (2.5 mM), chloroquine (10 µM), and transfected with ATG5 siRNA plasmid (2 µg). Accordingly, we conclude that hinokitiol inhibited cell proliferation in normal and tumor cells through different mechanisms, including modulating cell autophagy, cell cycle regulation, the p53-independent DNA damage response, and senescence.

### Effects of hinokitiol on tumor growth of H1975 cell xenografts in SCID mice

**Figure 7:**
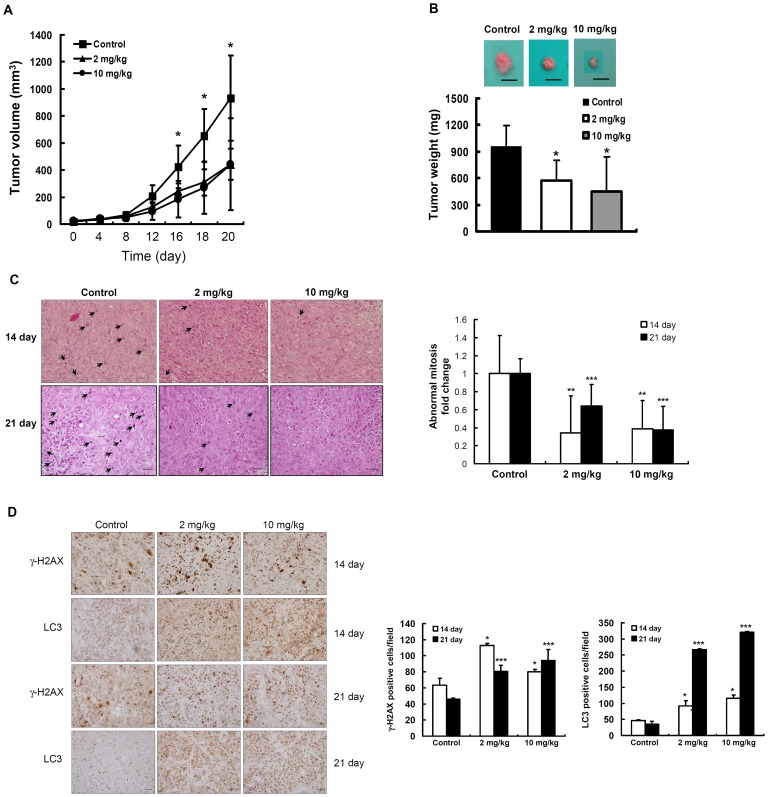
*In vivo* antitumor activity of hinokitiol. (**A**) The growth curves of subcutaneous xenografts of H1975 are shown. (**B**) The excised tumors were weighed and imaged. All results are given as the mean ± SD; n = 5 - 7 for each group. *indicates a significant difference at the level of *p*<0.05 compared with the control group. (**C**) Hematoxylin and eosin-stained tumor sections at days 14 or 21 from each group were analyzed. Arrow heads indicate the atypical nuclei or abnormal mitosis. Immunohistochemically stained tumor sections at days 14 or 21 from each group were analyzed to assess γ-H2AX and LC3 expression (**D**). The atypical nuclei, abnormal mitosis, and positive cells were quantified at 400× magnification under a standard light microscope (Olympus BX51, Japan). Each value is the mean ± SD of 5–10 fields of triplicate tumor sections. *, ** and *** indicate a significant difference compared with its' own control at the level of *p*<0.05, *p*<0.01, and *p*<0.001, respectively.

The *in vivo* antitumor activity of hinokitiol was evaluated using H1975 cell xenografts in NOD-SCID mice. The intra-peritoneal administration of hinokitiol at low (2 mg/kg/day) and high (10 mg/kg/day) doses for 21 days significantly reduced the tumor volume (47.58% and 47.59%, respectively; Fig. 7A) compared with the control group. The size and weight of the excised tumors showed that hinokitiol effectively inhibited tumor growth *in vivo* (Fig. 7B). The histological examination of the tumor sections revealed that hinokitiol was able to reduce abnormal mitosis compared with the control group at days 14 and 21 (Fig. 7C). To further investigate whether the tumor growth inhibition was related to DNA damage and autophagy, we confirmed the presence of γ-H2AX and LC3 expression in the tumor tissue using immunohistochemical staining. The histological analysis revealed that hinokitiol induced γ-H2AX and LC3 at days 14 and 21 (Fig. 7D) compared with the control group levels. These *in vivo* data suggest that hinokitiol reduced tumor growth, potentially through the attenuation of tumorigenicity, and induced DNA damage and autophagy to suppress tumor progression.

## Discussion

Natural herbs have been suggested as promising potential resources for the development of novel chemotherapeutics for cancer treatment. In this study, we assessed the effects of hinokitiol, which is also known as β-thujaplicin. Hinokitiol is the essential oil of a plant-derived, naturally occurring, aromatic, seven-membered tropolone compound found in cupressaceous plants such as the heartwood of *Chamaecyparis taiwanensis* and the leaves of *Calocedrus formosana* [22]. Hinokitiol has been reported to have applications in regulating several biological activities including anti-inflammation [23], anti-bacterial [24], anti-fungal [25], and anti-viral activities [26]. It has also been shown to have anti-proliferative effects in various cancer cell lines including melanoma [27], prostate carcinoma [28], oral cancer [22] and colon cancer cells [29]. However, the effects of hinokitiol on lung adenocarcinoma cells and the mechanisms underlying its effects have not been fully elucidated. In previous studies, the effective dose of hinokitiol against cancer cells ranged from 5 to 800 µM [27], [30]. Considering the bioavailability and the anti-proliferation evidence in this study, we selected 5 µM as the dose in the beginning. In addition, we determined the IC_50_ of hinokitiol in H1975 and PC9-IR cells are less than 2 µM. In order to determine the effect of hinokitiol on cell proliferation in series of cell lines, we used a wide range of doses, including a higher dose of 10 µM. For these reasons, we selected doses of 5 and 10 µM hinokitiol in the trypan blue staining test (Table 2) and a dose of 5 µM for the following experiments. The data showed that 5 µM could induce significant phenotypic changes under our conditions.

In this study, we demonstrated that hinokitiol significantly inhibited cell proliferation in a series of lung adenocarcinoma cell lines, including EGFR-mutant and TKI-resistant cells, H1975 and PC9-IR, respectively. The mechanism of H1975 resistance to gefitinib is due to T790M mutation, whereas that of PC9-IR, which was selected from parental PC9 cells that had been continuously exposed to increasing concentrations of gefitinib, could be associated with persistent activation of ERK pathway [18], [31]. In addition, the IC_50_ of gefitinib is more than 10 µM in H1975 [32] but more than 5 µM in PC9-IR, both of which contrast with 41 nM in the parental PC9 cells [18], [31].

DNA damage induction is an effective mode of action of anticancer agents. Anticancer agents act by producing sufficient DNA strand breaks in cancer cells to evoke cell repair systems, cell cycle arrest, or cell death programs [33], [34]. Many direct or indirect stresses lead to γ-H2AX expression, which is a sensitive central marker for DNA double-strand breaks (DSBs). These stresses including reactive oxygen species (ROS), DNA alkylation, topoisomerase poisons, repair deficiency, telomere shortening, meiosis breaks, and infection can activate ataxia telangiectasia, rad3-related (ATR), DNA-dependent protein kinase (DNA-PK) and ataxia telangiectasia mutated (ATM). ATM kinase is the major regulator of the recruitment of DNA damage response proteins to the DSB site and is considered a major mediator of γ-H2AX phosphorylation [35]–[37]. In addition to γ-H2AX, SMC3 is a substrate of ATM. SMC3 is a component of cohesin, which segregates the chromosome properly during S-phase, and its phosphorylation is required for DNA repair in response to DNA damage [37], [38]. Cohesin recruitment continuously enhances ATM and γ-H2AX phosphorylation [39]. In this study, we demonstrated that in lung adenocarcinoma cells, hinokitiol caused DNA damage by inducing DSBs. This observation was further supported by the increase in the phosphorylation levels of ATM, γ-H2AX, and SMC3. The induction of γ-H2AX by hinokitiol was further confirmed in the xenografts model. Moreover, XPC, ERCC1, and CRY1, which are involved in the DNA damage repair system and correlate with S-phase arrest and senescence, were also activated by hinokitiol. In addition, the accumulation of DNA damage was considered to be the major trigger of the cell senescence phenotype [40]. Accordingly, we suggest that hinokitiol induced cell cycle arrest in S phase and triggered senescence to prevent cell replication and the transmission of damaged DNA to daughter cells.

Presently, the molecular mechanism of hinokitiol-induced DNA damage is not fully understood. Although oxidative stress can cause DNA damage, we found that hinokitiol did not induce ROS generation in lung adenocarcinoma cells (data not shown). Metals have been demonstrated to play an important role in cell proliferation and survival, and metal-chelating agents can cause DNA damage and cell death in cancer cells [41]. Hinokitiol has metal-chelating activity, and in prostate carcinoma cells, it is able to inhibit the Fe-containing enzyme ribonucleotide reductase and disrupt zinc finger motifs, thus interfering with DNA synthesis and cellular activities [28]. We suggest that hinokitiol-induced DNA damage might be associated with its metal-chelating activity. Anticancer agents can induce DSBs, cell cycle arrest, and cell death via p53-dependent and -independent pathways [42], [43]. We demonstrated that DNA damage induced by hinokitiol was independent of p53, as demonstrated by the increased γ-H2AX expression without total or phosphorylated p53 activation in p53-wild-type H1975 cells and p53-null H1299 cells. Hinokitiol targeted cancer cells independent of their p53 status and can therefore be employed in a broad spectrum of tumors [43], [44]. Recent studies have shown that DNA damage signaling cascades are important inducers of autophagy, which maintains the balance between synthesis, degradation, and the recycling of cellular components process [45], [46]. In this study, hinokitiol induced autophagy, but not apoptosis or necrosis, in lung adenocarcinoma cells in vitro and in vivo, as demonstrated by LC3, ATG5, and p62 expression and AVO formation measurements. These data confirmed that hinokitiol treatment triggered autophagy and that autophagic flux was activated. Moreover, 3-MA pretreatment partially rescued the inhibition of cell growth induced by hinokitiol, implying that hinokitiol might induce autophagic cell death in lung adenocarcinomas. However, the precise mechanism by which DNA damage triggers autophagy in this context requires further study. In addition, autophagy was identified as an effector mechanism that regulates senescence [47], [48]. In this study, hinokitiol-triggered senescence was attenuated when autophagy was chemically or genetically down-regulated. These data provided new insight that autophagy might regulate senescence and consequently suppress cell progression and limit tumorigenesis. However, the detail mechanism of how hinokitiol triggered senescence without inducing DNA damage or autophagy response in lung stromal fibroblasts is unclear and further investigations are needed. In addition, previous studies have reported that sudden changes of culturing conditions are a stress to trigger senescence [49]. In this study, the stromal fibroblasts dissect from human lung have to adapt to artificial environments, as well as the absence of surrounding cell types and extracellular matrix components in culture dishes. This inadequate culture condition might offer a potential explanation of why stromal fibroblasts are more sensitive to hinokitiol induced senescence. The other possibility could be due to that unlike the tumor cell lines, the normal stromal fibroblasts are not immortalized.

The impairment of cell cycle progression is one of the mechanisms of anticancer agents [50]. The protein cyclin E2, which is essential for the transition from G1 to S phase [51], was slightly induced by hinokitiol even though other cell cycle check point regulators were down-regulated. The cell cycle analysis by PI staining and BrdU incorporation assays consistently suggested that hinokitiol inhibited the proliferation of cells by arresting the cell cycle in S phase. In addition, the down-regulation of EGFR expression and the inhibition of EGFR signaling cascades, such as the RAS/MAPK, PI(3)K/Akt, PLCc/PKC, and Jak/STAT pathways, offer potential therapeutic strategies for inhibiting cell proliferation [2], [6]. We showed that hinokitiol inhibited EGFR phosphorylation and reduced ERK expression, which offers a possible mechanism by which hinokitiol suppressed proliferation in H1975 cells.

In this study, although the proliferation of stromal fibroblasts was also inhibited by hinokitiol treatment (data not shown), we found that hinokitiol induced DNA damage, autophagy, and cell cycle arrest to a greater extent in lung cancer cells than in stromal fibroblasts. The possible mechanisms of selectivity may be due to the following: 1) aneuploidy, which is a hallmark of cancer, increases the efficacy of anticancer agents [52]; 2) tumor cells are frequently more sensitive to Fe than normal cells and thus are more vulnerable to metal-chelating agents [28]; 3) the acidic environment in tumors enhances the growth inhibition and DNA fragmentation induced by metal-chelating agents [41]; 4) cancer cells with abundant topoisomerase 2α (Top2α) expression are more sensitive to DNA breaks induced by Top2α inhibitors [30]; 5) either the bioavailability or sensitivity of the drug towards certain biological targets in the cells may be altered [53]; 6) defects in the repair systems of tumor cells may increase their vulnerability [33]; or 7) in solid tumors, anticancer agents may enhance oxidative stress under hypoxic conditions [54].

In this study, we selected NOD-SCID mice as the xenograft model, based on previous studies [55], [56]. Tumorigenesis studies revealed that the presence of atypical nuclei indicates higher tumorigenicity or malignancy [57], [58]. The size and weight of tumors were obviously lower, and the histological examination revealed fewer abnormal mitosis or atypical nuclei in treated mice. The IHC data indicated that the higher expression levels of γ-H2AX and LC3-II in hinokitiol-exposed mice might suppress tumor progression, resulting in the inhibition of tumor growth [59], [60]. In addition, Shimizu et al. indicated that the acute oral LD_50_ for hinokitiol is as high as 469–504 mg/kg for mice [61]. Both 2-year chronic and carcinogenic toxicity studies have indicated that at dietary doses of 20.9–25.9 mg/kg/day in rats, hinokitiol does not have significant toxicity [62]. In this study, the mice in the hinokitiol group maintained a normal weight throughout treatment, and did not show any abnormalities with respect to food intake. Furthermore, hinokitiol treatment did not produce any severe adverse effects or life-threatening toxicities, as monitored by animal survival and behavior. Taken together, our data support the idea that hinokitiol might be used as a novel and safe strategy for the treatment of lung adenocarcinoma.

## Conclusions

**Figure 8:**
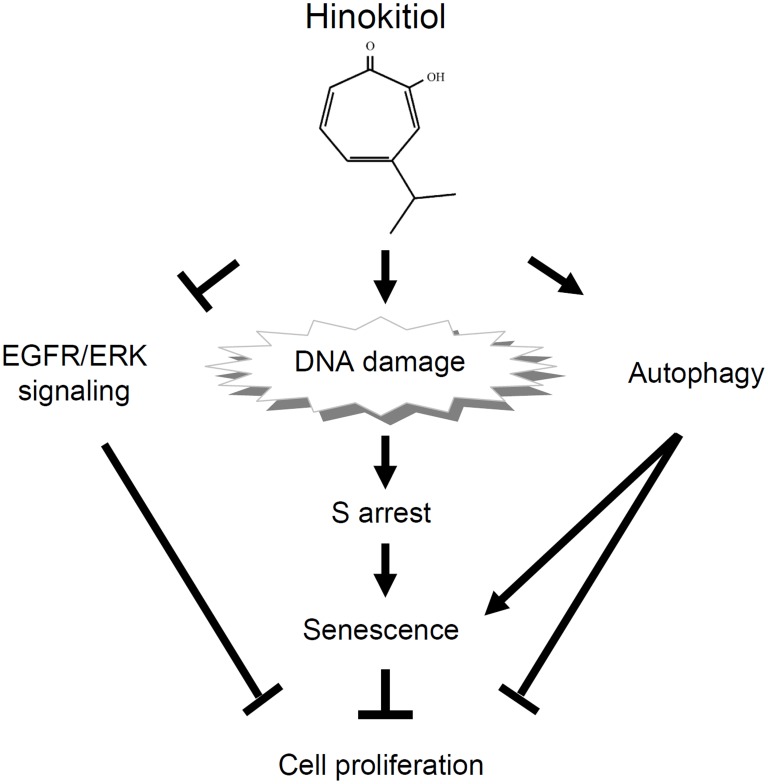
A schematic representation of the hypothetical mechanisms for the role of hinokitiol in suppressing lung adenocarcinomas.

This study reports, for the first time, that hinokitiol, isolated from *Calocedrus formosana* heartwood, possesses potent anticancer effects against lung adenocarcinoma cells via the induction of DNA damage, autophagy, cell cycle arrest, and senescence, as depicted in Fig. 8. Its antitumor activity *in vivo* occurred without weight loss or other life-threatening toxicities to the animal, supporting the potential of this naturally occurring compound as a candidate therapeutic agent in lung cancer treatments.
